# Regulation of neuronal transcription by RNA-binding proteins via R-loop dynamics

**DOI:** 10.4103/NRR.NRR-D-25-01373

**Published:** 2026-01-27

**Authors:** Shruti Singh Kakan, Ximena Corso-Díaz

**Affiliations:** Department of Ophthalmology, Byer’s Eye Institute, Stanford School of Medicine, Stanford University, Palo Alto, CA, USA

Neuronal cells require precise and stable control of gene expression throughout their extended lifespan, which presents numerous challenges for gene transcription. While traditional studies of transcriptional regulation have primarily focused on canonical DNA-binding factors, there is an increasing recognition of the role of regulatory RNAs and RNA-binding proteins (RBPs) in modulating this process. A wide repertoire of RNAs is expressed in eukaryotic cells, including many forms of non-coding RNAs that are transcribed from most genomic regions (Djebali et al., 2012). The majority of these non-coding RNAs play crucial roles in genome regulation, functioning both as products and regulators of gene transcription.

RNA regulation involves a conserved family of RBPs that participate not only in RNA metabolism but sometimes function as DNA-binding proteins, metabolic enzymes, transmembrane proteins, and E3 Ubiquitin ligases (Liao et al., 2025). Over 3000 human RBPs have been identified through experimental methods like RNA interactome capture and computational predictions (Liao et al., 2025). However, the functions of many remain largely unknown, with a significant number lacking canonical domains, indicating diverse and yet uncharacterized RNA interaction mechanisms. Notably, some RBPs are implicated in chromatin regulation and gene transcription, highlighting their roles that extend beyond RNA metabolism. RBPs widely localize to gene promoters and interact with the RNA polymerase II (Pol II) complex, chromatin remodelers, and histone modifiers to regulate euchromatin and heterochromatin, as well as the formation of biomolecular condensates that compartmentalize chromatin activities (Chen et al., 2019). Chromatin-associated RNAs and RBPs also mediate the complex long-range interactions that shape the three-dimensional genome architecture. The transcriptional regulator CCCTC-binding factor (CTCF) binds to RNA and RBPs and serves as an organizer of the chromatin structure (Wulfridge et al., 2023; Dehingia et al., 2025). Thus, RBPs play key roles beyond RNA metabolism, influencing chromatin regulation, gene transcription, and genome organization, highlighting the need to further explore their diverse functions and mechanisms of action.

**RNA-binding protein-mediated regulation of R-loops:** R-loops are dynamic triple-stranded structures comprised of DNA-RNA hybrids that form when the transcribing nascent mRNA hybridizes with its template DNA strand, displacing the complement strand as single-stranded DNA. Under certain sequence and torsional contexts, R-loops are structurally more stable than DNA-DNA duplexes (García-Muse and Aguilera, 2019). Long thought to be a byproduct of transcription, R-loops are now recognized as key regulators of gene expression, influencing transcription elongation, termination and promoting antisense transcription. R loops also engage in shaping the three-dimensional chromatin architecture by influencing the binding of the structural protein Cohesin and of CTCF to DNA (Wulfridge et al., 2023); and regulate the epigenome by controlling the methylation of DNA and histone H3 on lysine 9 (H3K9me2) (García-Muse and Aguilera, 2019; Wulfridge et al., 2023).

**Figure 1:**
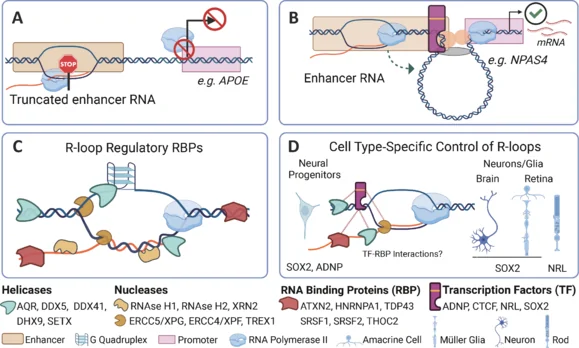
Mechanisms of R-loop homeostasis in neuronal cells. R-loops are triple-stranded structures formed during transcription, consisting of an RNA-DNA hybrid and a single-stranded DNA. The G-rich displaced strand can form G-quadruplexes (G4s), which promote the formation and stabilization of R-loops. Cell type-specific R-loop formation is critical for the regulation of neuronal genes and can have opposite effects on gene expression. For example, Pol II is halted by an R-loop at the enhancer of (A) APOE (Watts et al., 2022), resulting in the generation of a truncated enhancer RNA that prevents gene activation. R-loops can also activate transcription by facilitating interactions between enhancers and promoters through chromatin looping as in (B) NPAS4 (Akiki et al., 2024). (C) Several RBPs control R-loop formation and resolution and many were shown to exert their effects in neuronal cells. Nucleases, including RNase H1 cleave the RNA of the R-loop to prevent excessive accumulation of hybrids, or to initiate DNA replication in the mitochondria (Brickner et al., 2022). RNA helicases can remove the RNA from the hybrid and a few, such as DDX1 and DHX9, are also implicated in R-loop formation (Yang et al., 2023). Various multifunctional RBPs without helicase activity and roles in mRNA splicing and export are also involved in R-loop regulation. (D) A candidate mechanism for cell-specific regulation of R-loops involves the binding of cell type-specific transcription factors to R-loops and RBPs. For example, NRL, the master regulator of the transcriptional program of retinal rod photoreceptors, interacts with R-loops and a plethora of RBPs, including DHX9 (Corso Diaz et al., 2025). ADNP interacts with R-loops and is critical for their resolution in early neural development (Wulfridge et al., 2023). The DNA and RNA binding TF Sox2, known to maintain neural stem cell identity, regulates the proliferation, maturation, and maintenance of Müller glia and a subset of amacrine cells, and has been shown to interact with Ddx5 to mediate cell reprogramming through R-loops (Li et al., 2020). Created in BioRender. Singh Kakan, S. (2025) https://BioRender.com/51fh71p. ADNP: Activity dependent neuroprotector homeobox; APOE: apolipoprotein E; AQR: aquarius intron binding spliceosomal factor; ATXN2: ataxin 2; CTCF: CCCTC-binding factor; DDX41: DEAD-box helicase 41; DDX5: DEAD-box helicase 5; DHX9: DExH-box helicase 9; ERCC4: ERCC excision repair 4, endonuclease catalytic subunit; ERCC5: ERCC excision repair 5, endonuclease; HNRNPA1: heterogeneous nuclear ribonucleoprotein A1; NPAS4: neuronal PAS domain protein 4; NRL: neural retina leucine zipper; RBP: RNA-binding protein; SETX: senataxin; SOX2: SRY-box transcription factor 2; SRSF1/2: serine and arginine rich splicing factor 1/2; TDP-43: TAR DNA binding protein; THOC2: THO complex subunit 2; TREX1: three prime repair exonuclease 1; XRN2: 5’’-3’’ exoribonuclease 2.

A diverse array of mechanisms exists to regulate R-loop formation, with novel R-loop interactors continually being identified (**Figure 1**). RBPs belonging to a multitude of families are frequently found in R-loop proteomes (Yang et al., 2023) and may have direct roles in regulating their resolution. RBPs also coordinate nascent RNA processing (e.g., splicing) with transcription, ultimately influencing R-loop formation (García-Muse and Aguilera, 2019). Nucleases such as RNase H1 and H2 facilitate the degradation of the RNA component of R-loops, and RNA helicases, particularly SETX, DHX9, and DDX5, are crucial for unwinding R-loops (Yang et al., 2023). Disruptions in these RBPs or their interactions can lead to R-loop dysregulation (Yang et al., 2023). Thus, RBPs play a crucial role in the formation and resolution of R-loops to ensure proper gene expression.

**Figure 2:**
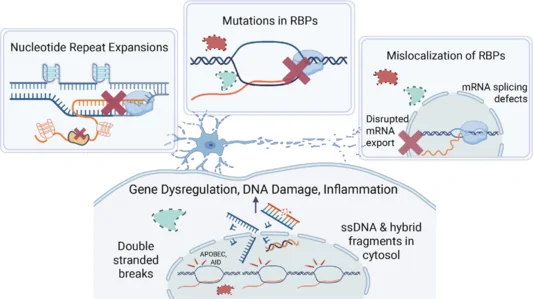
Mechanisms of R-loop dysregulation in neurodegeneration. Various mechanisms can lead to the accumulation of harmful R-loops. Insertion of trinucleotide or hexanucleotide repeat expansions forms G-quadruplexes that stabilize hybrids and hinder the binding of R-loop regulators. This, in turn, can lead to gene silencing (e.g., C9orf72 or FXN), and anti-sense or bidirectional transcription (Brickner et al., 2022; Beghѐ et al., 2025). Mutations in RBPs such as SETX or TDP-43 can create dysfunctional proteins or alter their cellular localization, resulting in aberrant RNA metabolism, global R-loop accumulation, and disruption of Pol II-mediated transcription (Beghѐ et al., 2025). This potentially affects long genes and may hamper neuronal cell-specific functions such as synaptic transmission (Beghѐ et al., 2025). Accumulated R-loops can be exposed to nucleases, deaminases or ROS for extended periods, which can trigger DNA breaks, spilling gDNA, or hybrid fragments into the cytosol and triggering an inflammatory response (Brickner et al., 2022). Created in BioRender. Singh Kakan, S. (2025) https://BioRender.com/go7s7q. AID: Activation induced cytidine deaminase; APOBEC: apolipoprotein B MRNA editing enzyme; C9orf72: C9orf72-SMCR8 complex subunit; FXN: frataxin; gDNA: genomic DNA; Pol II: RNA polymerase II; RBP: RNA binding protein; ROS: reactive oxygen species; SETX: senataxin; ssDNA: single stranded DNA; TDP-43: TAR DNA binding protein.

Aberrations in R-loop homeostasis can cause their accumulation, leading to genome instability and diseases such as neurodegeneration and cancer (Brickner et al., 2022; Beghѐ et al., 2025; **Figure 2**). R-loops can accumulate in many ways; for example, when the RBPs that regulate them become dysfunctional, or when DNA mutations increase GC skew, leading to the formation of highly stable alternative structures. Notably, insertion of G-rich nucleotide repeat expansions form R-loops that can be stabilized by G4-quadruplexes on the complementary strand, and are seen in patients with amyotrophic lateral sclerosis, Huntington’s disease and various ataxias (Brickner et al., 2022; Beghѐ et al., 2025). In mitotic cells, R-loops can cause replication-transcription conflicts (Brickner et al., 2022), and lead to DNA damage (Jackson et al., 2025) and hyper-recombination (García-Muse and Aguilera, 2019). Accumulated R-loops can also expose the complementary single-stranded DNA to nucleases, reactive oxygen species, and cytosine deamination through APOBEC, thus increasing the risk of DNA damage, de-novo mutations, and ultimately triggering an inflammatory response (Beghѐ et al., 2025; Jackson et al., 2025).

**Role of R-loops in neuronal cells:** Gene transcription presents many challenges in neuronal cells due to their extended lifespan and requirement for activity-dependent gene expression. Additionally, neurons have a high metabolic rate and express longer genes, which increase their susceptibility to genomic instability and DNA damage. Together, this makes neurons vulnerable to the loss of R-loop homeostasis. Intriguingly, R-loops are more frequently observed on long genes in the retina (Corso Diaz et al., 2025), presenting a potential mechanism for neuronal-specific gene regulation. Indeed, R-loops accumulate in long genes (such as those involved in synaptic function) in Cockayne syndrome and contribute to the disease by stalling Pol II during transcriptional elongation in neuronal cell models (Brickner et al., 2022; Beghѐ et al., 2025). Mouse neural progenitors reportedly have 27 highly evolutionarily conserved long genes (> 100 kb) involved in neuronal junction adhesion or synapses, with a high susceptibility for recurrent double strand breaks that are reliant on XRCC4 for repair (Wei et al., 2016). Most of these genes (21/27) harbor R-loops in mouse rod photoreceptors (Corso-Diaz et al., 2025). This suggests a potential role for R-loop resolution in the maintenance, stable expression and/or genomic integrity of long neuronal genes. Notably, recent evidence is also emerging on the role of R-loops in neuronal enhancer function (**Figure 1**). An R-loop formed at an enhancer for the immediate-early response gene neuronal PAS domain protein 4 (*Npas4*) was shown to be required for behavioral adaptations produced by chronic psychosocial stress or cocaine exposure in mice (Akiki et al., 2024). Similarly, an enhancer R-loop formed by the eRNA AANCR was shown to regulate the transcription of apolipoprotein E (*APOE*) (Watts et al., 2022). In this instance, the mechanism involves RNA abasic sites within the R-loop, which stalls Pol II and inhibits APOE expression; this inhibition is overridden during cellular stress. Genetic variation at this enhancer correlates with APOE expression in the hippocampus and may contribute to the pathogenesis of Alzheimer’s disease (Watts et al., 2022). Similarly, in the mouse retina, R-loops localize to intergenic regions harboring histone marks associated with enhancers (Corso Diaz et al., 2025). Thus, new studies are needed to shed light into the diverse roles of R-loops in the complex regulation of neuronal genes.

R-loops contribute to the pathology of many neurodegenerative diseases and are associated with multiples hallmarks of neurodegeneration, including transcriptional dysregulation, repeat instability, protein aggregation, inflammation, mitochondrial stress, DNA damage, and neuronal cell death (Beghѐ et al., 2025). The importance of R-loops in the nervous system is underscored by the role of numerous RBPs linked to neurological disorders that are involved in R-loop resolution (**Figures 1** and **2**). RBPs such as TAR DNA-binding protein 43 (TDP-43), fused in sarcoma (FUS), senataxin (SETX), and aquarius (AQR), which are linked to amyotrophic lateral sclerosis and various ataxias, function in R-loop homeostasis (García-Muse and Aguilera, 2019; Beghѐ et al., 2025). Additionally, mutations in these RBPs can alter their localization and prevent their recruitment to active Pol II sites to resolve R-loops in time. Stuck R-loops can be cleaved by the endonucleases XPG (ERCC5) and XPF (ERCC4) that generate DNA breaks and a subsequent inflammatory response (Beghѐ et al., 2025). Similarly, several members of the Transcription Export (THO/TREX) protein complex, involved in mRNA nuclear export, are critical regulators of R-loop removal (García-Muse and Aguilera, 2019; Beghѐ et al., 2025). Loss of TREX1 function causes the neuroinflammatory disease Aicardi-Goutières syndrome (Beghѐ et al., 2025). Thus, R-loops play a critical role in neuronal gene regulation, and the intricate relationship between R-loops, RBPs, and gene expression highlights their potential as key players in the pathogenesis of multiple neurodegenerative disorders.

**The interplay of RBPs, transcription factors, and R-Loops:** Most RBPs are ubiquitously expressed, yet how they exert cell-type-specific functions is largely unknown. Emerging examples illustrate how transcription factors (TFs) and their cofactors interact with RBPs, imparting specificity and influencing chromatin dynamics. Given that transcription factors widely interact with RNA, it is unsurprising that TF/RBP complexes play a pivotal role in regulating chromatin activity (**Figure 1**). One notable example of TFs interacting with RBPs occurs within R-loops. The TF Sox2, for instance, interacts with the RBP Ddx5, inhibiting its R-loop-resolvase activity during somatic cell reprogramming (Li et al., 2020). Similarly, the rod photoreceptor-specific transcription factor Neural Retina Leucine Zipper (NRL) shows extensive interactions with RBPs and R-loops in the mouse retina (Corso Diaz et al., 2025). The interaction between NRL and DHX9, observed in both human and mouse retinas, may be crucial for NRL’s regulation of rod-specific transcriptional programs through R-loops, as evidenced by the co-localization of NRL and R-loops at numerous genomic loci (Corso Diaz et al., 2025). R-loops also co-localize with CTCF at various genomic regions in mouse embryonic stem cells and mouse retina, and together with G4 quadruplexes, R-loops are necessary to stabilize and enhance CTCF binding to its DNA motif *in vitro* (Wulfridge et al., 2023; Corso Diaz et al., 2025). Activity Dependent Neuroprotector Homeobox (ADNP), a neuroprotective TF, is essential for neural differentiation. ADNP regulates CTCF binding to chromatin during early embryo neurogenesis and suppresses R-loop formation in a site-specific manner *in vitro* and *in vivo* (Yan et al., 2022). Intriguingly, this R-loop resolution activity was found to be independent of ATP hydrolysis. In iPSC neurons derived from patients with Autism spectrum disorder, a dysfunctional variant of ADNP led to R-loop accumulation, highlighting the importance of proper R-loop resolution in neuronal differentiation and neocortex development (Yan et al., 2022). A recent study demonstrated that chromatin interactions and insulating regions known as topologically associated domains boundaries progressively strengthen during the differentiation of embryonic stem cells into the neural lineage (Dehingia et al., 2025). This process was shown to be mediated by increased interactions in G4 rich regions between CTCF and RBPs, many of which are regulators of R-loops, including DDX5. Thus, TFs may regulate cell type-specific programs by modulating R-loops through interactions with specific RBPs or by mechanisms that are not yet fully understood. This underscores the need for further studies on the interplay between TFs, chromatin architecture, R-loops, and RBPs.

**Conclusions and future directions:** The intricate interplay between RBPs, transcription factors, and R-loops highlights a complex regulatory network that is essential for gene expression in neuronal cells. Emerging evidence reveals the multifaceted roles of RBPs that extend beyond RNA metabolism to include chromatin regulation, transcriptional control, and genome organization. RBP interactions with cell type-specific molecules are pivotal for maintaining cellular homeostasis and responding to environmental stimuli. Future research should aim to elucidate the specific mechanisms by which RBPs, and TFs coordinate R-loop dynamics, particularly in the context of neuronal gene regulation, chromatin architecture, enhancer activity and the pathogenesis of neurodegenerative diseases. Importantly, R-loops may represent mutation-prone regions, as recently demonstrated in de novo datasets for rare diseases (Jackson et al., 2025), making their study crucial for understanding human pathologies. R-loops have emerged as potential therapeutic targets for neurodegenerative diseases and can be targeted with antisense oligonucleotides and Clustered Regularly Interspaced Short Palindromic Repeats technologies (Akiki et al., 2024; Beghѐ et al., 2025). However, given their complex and multifaceted roles in various cellular processes, it is crucial to understand their interactions with cell type-specific factors to expand the number of therapeutic targets and improve the specificity of therapies. An important avenue for further research involves exploring additional TFs and RBPs that participate in R-loop formation or resolution in neurons. Numerous RBPs have been identified within R-loop proteomes and may influence R-loop dynamics in various ways, such as binding to single-stranded DNA through their RNA-binding domains, or be recruited to R-loops through their intrinsically disordered regions. Notably, analyses in neuronal cells, including retinal cells, are essential for uncovering novel R-loop regulatory mechanisms, especially given their remarkable diversity of RNA species, and elevated expression levels of various RBPs, including those associated with R-loops (Corso Diaz et al., 2025). These studies are also significant in the context of neural differentiation and potentially regeneration, as R-loops may play a key role in maintaining the regulation of long neuronal genes involved in synapse and cell-cell adhesion. In addition, RBPs and R-loops shape the chromatin architecture necessary for differentiation into neural fates, for example, through their interaction with ADNP and CTCF. Furthermore, somatic cell reprogramming, which is important for advancing cell therapy, also relies on proper R-loop regulation through interactions between RBPs and TFs as demonstrated by Sox2 and Ddx5. Understanding the complex interplay among RBPs, regulatory RNA, TFs, and R-loops may provide insights into the organization of the functional genome. This knowledge could reveal novel therapeutic targets and strategies for modulating neural gene expression programs and mitigating the effects of R-loop dysregulation in neurodegenerative diseases.

*This work was supported by the National Eye Institute, grant number R00EY030918 and the Association for Research in Vision and Ophthalmology (ARVO) Genentech Career Development Award to XCD.*

**Additional file:** *Open peer review report 1.*

OPEN PEER REVIEW REPORT 1
