# Supplementary material for: Regulation of neuronal transcription by RNA-binding proteins via R-loop dynamics
Source: Neural Regen Res. 2026 Jan 27;21(10):4878–9. doi: 10.4103/NRR.NRR-D-25-01373 (PMC13568653; doi:10.4103/NRR.NRR-D-25-01373)
Supplement: Supplementary file 1 [file NRR-21-4878_Suppl1.pdf]

OPEN PEER REVIEW REPORT 1

Name of journal: Neural Regeneration Research
Manuscript NO: NRR-D-25-01373
Title: Regulation of Neuronal Transcription by RNA-Binding Proteins via R-Loop Dynamics
Reviewer's Name: Yuliang Wu
Reviewer's country: Canada

COMMENTS TO AUTHORS

In this short Perspectives, authors discussed "Regulation of Neuronal Transcription by RNA-Binding Proteins via R-Loop Dynamics". I thought 3 sections are well arranged: RBP-mediated regulation of R-loops, The role of R-loops in neuronal cells, and the interplay of RBPs, transcription factors, and R-Loops. The topics are welcome in the field, and the time is good.

Here are some issues that I found when I read it:

1. Page 2 (no page number?) line 16: Eukaryotic, lowercase e
2. Page 2 line 14-30:" A wide diversity of RNAs is expressed in Eukaryotic cells, as revealed by high-throughput sequencing technologies that have revolutionized our understanding of mammalian genomes. These advancements have uncovered an extensive level of transcriptional activity that generates long non-coding RNAs (lncRNAs), repeat-derived RNAs, and enhancer RNAs (eRNAs), among others (Djebali et al., 2012). Protein-coding genes also contribute to this complexity by producing various smaller RNA species (Djebali et al., 2012). Most regulatory RNAs remain in the nucleus, playing crucial roles in genome regulation. Thus, RNA serves as both a product and a regulator of gene transcription."
It is very close to a published paper in terms of content and structure:
"The advent of deepsequencing technologies has now revealed that mammalian genomes are far more active in transcription (Djebali et al. 2012), generating a large repertoire of regulatory RNAs, including long noncoding RNAs (lncRNAs) (Long et al. 2017), repeat-derived RNAs (Johnson and Straight 2017), and enhancer RNAs (eRNAs) (Li et al. 2016). Even protein-coding genes are producing various smaller RNA species that are either cause or consequence of regulated gene expression as a result of divergent or convergent transcription and transcription pausing and pause release (Wissink et al. 2019). Most regulatory RNAs are predominantly retained in the nucleus (Li and Fu 2019), where they may modulate gene expression at different steps of transcription on specific transcription units or genomic loci (Skalska et al. 2017), remodel chromatin structures and dynamics (Bohmdorfer and Wierzbicki 2015), and mediate long-distance genomic interactions (Schoenfelder and Fraser 2019), together contributing to the organization of the three-dimensional (3D) genome." Chen et al, Cold Spring Harb Symp Quant Biol. 2019:84:55-66.
3. Page 2 line 32-36: "not only in RNA metabolism but also function as DNA-binding proteins, metabolic enzymes, transmembrane proteins, and E3 Ubiquitin ligases (Liao et al., 2025)", needs re-word, because it sounds like all RBPs can bind DNA…
4. "Chen et al., 2019" was cited heavily, for example from page 2 line 47-page 3 line 25, every sentence has cited Chen 2019, except 1 from Saldana-Meyer et al., 2019, which is problematic, because the current manuscript is a Perspectives, not a commentary on Chen 2019. Moreover, "Chen et al., 2019" is a review paper that might summarize relevant results 6-10 years ago, no new finding in recent years?
5. Page 3 line 36: "coding RNA such as guide RNAs, eRNA and antisense lncRNA (Santos-Pereira and Aguilera, 2015)", guide RNA was not mentioned in that paper, eRNA was not mentioned as forming R-loop in that paper. Again, Ref (and knowledge) issue, "Santos-Pereira and Aguilera, 2015" was cited heavily on page 3 line 34-52, it was a review 10 years ago! In addition, original research articles are preferred.
6. Page 3 "R-loops are now recognized as key regulators of gene expression, influencing transcription elongation, termination and promoting antisense transcription. R loops also engage in shaping the 3D

chromatin architecture by influencing Cohesin and CTCF binding (Santos-Pereira and Aguilera, 2015; Wulfridge et al., 2023); and regulate the epigenome by controlling the methylation of DNA and histone H3 on lysine 9 (H3K9me2) (Santos-Pereira and Aguilera, 2015)." This should be developed, as it is the key of this Perspectives: how RBPs (via R-loops) regulate transcript in neurons?
7. G-quadruplexes (G4s) should be mentioned and discussed in main text, not only in Figure legend.
8. Figure 1:
* a and b, the cartoon did not show clearly HOW R-loop inhibits the transcript of APOE (a) and promote NPAS4 (b)
* c, other RBP should not bind the displaced ssDNA. Might sperate the nuclease, as RNase H1/2 target ssRNA present in the RNA:DNA hybrid, XPG (ERCC5) and XPF (ERCC4) target dsDNA or RNA:DNA hybrid, and XRN2 is a 5′ to 3′ exoribonuclease on ssRNA. ATXN2 and TDP43 are not helicase. What does the scissor stand for?
* d, should be improved, at least the figure itself did not show anything cell-type specific. What is the left cartoon meaning? Other RBPs and TFs would be better in different colors.
9. Figure 2 should be improved as well. I like the neuron in the middle, but I did not get much about the 4 surrounding images. According to the title: "R-loop dysregulation in neurodegeneration", I thought it would be good to show it in subsequent steps: such as RBP-R-loop/G4-transcription-neuronal cell functions- neurodegeneration diseases. I am ok to cite Ref in Figure legend, but both Figure and Figure legend should stand alone—should be understood without looking into main text and Ref. This applies to Figure 1 as well.
10. Lastly, as the journal titled with "Neural Regeneration", any more and deep discussion related to neural regeneration will be appreciated.
